# Why British Public Sanatoria Have Failed

**Published:** 1917-10-20

**Authors:** 


### WHY BRITISH PUBLIC SANATORIA HAVE FAILED.

One effect of war conditions has been increasingly to undermine the efficiency of British sanatoria to an extent which is not yet realised by the public nor probably by the profession.

Readers of The Hospital will recall the mention in our columns from time to time of the precarious tenure of posts as superintendents of public sanatoria. The circumstances of yet another resignation have been recently communicated to us, as set forth in a report by the public health authorities in a south-eastern county. The temporary (non-resident) superintendent of a sanatorium there resigned because his refusal to re-admit five out of seven discharged patients was not accepted by the Insurance Committee responsible for these patients. In the sequel this latter body and the Public Health Committee held a joint meeting and decided—*inter alia*—that the Insurance Committee would not accept complaints from patients unless first satisfied that such complaints had been preferred to those in authority at the sanatorium; or, in the event of these officers failing to remove the cause of the complaints, until the same had been investigated by the Hospitals Sub-Committee. Upon these and some other conditions it seemed likely that the superintendent and staff would continue to manage the institution for the duration of the war. We congratulate the parties concerned on the settlement of the dispute, more especially as war conditions seem to have been at the bottom of the whole affair.

In his published statement the medical man concerned explained that he could only give part of his time to the management of the sanatorium, which management, he said, accordingly devolved chiefly upon women. The inadequacy of such an arrangement is probably responsible for the great numbers of patients, especially military patients, now leaving sanatoria all over the country against medical advice. Convalescent men patients will not obey women with the particularity which the hygienic-dietetic treatment of tuberculosis demands in order to be at all efficient. But, like so much else, the management of our public sanatoria needs thorough reconstruction.

The hospital system, which means that the resident medical men have not much more than the position of house physicians, has been proved for years past to be wholly unsuitable. What are wanted are large central institutions with surrounding land developed fully for farm work, with a supreme resident medical man of mature age and experience and clinical ability, thus closely resembling in constitution our asylums for the insane. Then there would be an end of the constant squabbles and complaints and harmful lapses in continuity of treatment, with which those having inside knowledge of British public sanatoria are familiar.

